# Building Trust in a Newly Established Physician–Patient Relationship: A Scoping Review

**DOI:** 10.1111/hex.70788

**Published:** 2026-07-27

**Authors:** Barbora Mechúrová, Šárka Pomichálková, Janka Šefranková, Martin Loučka

**Affiliations:** ^1^ First Faculty of Medicine Charles University Prague Prague Czech Republic; ^2^ Third Faculty of Medicine Charles University Prague Czech Republic; ^3^ Department of Medical Psychology and Ethics, Faculty of Medicine Masaryk University Brno Czech Republic

**Keywords:** communication, patient‐centred care, physician–patient relations, scoping review, trust

## Abstract

**Background:**

Trust is a fundamental component of the physician–patient relationship, influencing cooperation, adherence, and patient outcomes. While much research has focused on long‐term relationships, little is known about the factors that shape trust during the very first physician–patient encounter.

**Objective:**

The aim of this study was to assess the factors contributing to the development of patients' trust in a newly established physician–patient relationship.

**Search Strategy:**

We conducted a scoping review and applied thematic analysis to synthesise findings from identified studies. PubMed database was searched using predefined keywords for articles published between January 1975 and January 2026.

**Data Extraction and Synthesis:**

Initially, 2825 studies were identified, with 2818 remaining after duplicates were removed. Studies underwent title and abstract screening, with 142 shortlisted based on inclusion criteria. Studies were eligible if they focused on the first physician–patient encounter and trust was either the primary evaluated variable or one of the evaluated variables. Full‐text assessments reduced this to 10 studies for thematic analysis.

**Results:**

Factors positively associated with trust building were organised according to the phase in which they appeared to influence trust: pre‐consultation, during consultation, and post‐consultation. In the pre‐consultation phase, physician reputation, care coordination, and previous positive experiences appeared to support trust. During consultation, a warm approach, perceived technical skills, information sharing, patient participation, active listening, and empathy enhanced trust. Post‐consultation, trust was reinforced by symptom relief and recovery. An apology for medical errors was crucial as well. Conversely, limited opportunities to ask questions, time constraints, unmet expectations, stigmatisation, and inappropriate language appeared to be negatively associated with trust.

**Discussion and Conclusions:**

The findings suggest that trust building begins before the initial encounter, continues throughout the consultation itself, and may be reinforced by experiences following the visit. Trust between physicians and new patients appears to be shaped by both technical competence and communication skills. Medical education should therefore encompass both domains to support high‐quality care and effective clinical relationships.

**Abbreviations**
CASP: critical appraisal skills programme
GSE‐6: general self‐efficacy scale – 6 item version
HIV: human immunodeficiency virus
ROBINS‐I: risk of bias in non‐randomised studies of interventions
STAI: state‐trait anxiety inventory
UK: United Kingdom
VA: veterans affairs

## 1 Introduction

The growing emphasis on patient‐centred care has highlighted the importance of the physician–patient relationship as a fundamental component of high‐quality care. Physician's interpersonal skills are no longer viewed merely as the ‘icing on the cake’ of medical competence but as a vital component of effective healthcare delivery [1]. Together with professional expertise, these skills shape the quality of interactions between physicians and patients and may contribute to the development of trust within therapeutic relationships [2, 3].

Trust is widely recognised as a multidimensional construct, although no single definition has achieved universal acceptance within the literature. In healthcare, trust is commonly conceptualised as the acceptance of a vulnerable situation in which patients believe that healthcare professionals will act in their best interests [4, 5]. Central to most conceptualisations are the notions of vulnerability, uncertainty, dependence on another person, and positive expectations regarding that person's future actions [2, 4, 5, 6, 7].

Trust in physicians is generally regarded as a multidimensional construct shaped by several interrelated domains. Thom et al. [4] describe three principal components influencing trust: technical competence, interpersonal competence, and agency. Technical competence refers to physicians' professional knowledge and clinical expertise, whereas interpersonal competence encompasses communication skills, empathy, honesty, and respectful behaviour. Agency refers to the belief that physicians prioritise patients' welfare and act in their best interests when making clinical decisions [4, 8]. Similarly, Hall et al. [5] argue that patients place trust not only in physicians' professional competence but also in their human qualities, moral integrity, and ethical values [5].

Evidence suggests that trust within the therapeutic relationship influences several important patient outcomes. Higher levels of trust are associated with greater cooperation, reduced fear and uncertainty related to the diagnostic process [9], increased patient involvement in shared decision‐making [10], better adherence to recommended treatment [11, 12, 13, 14, 15, 16, 17], greater continuity of care [18, 19], and a lower likelihood of seeking a second opinion [20]. Conversely, patients who perceive an unsatisfactory affective relationship with their clinician, including an inability to trust the clinician and the perception that they are not being treated as ‘a person,’ are more likely to disregard medical advice regarding attendance at recommended follow‐up appointments [21] and less likely to report symptom improvement [22]. Although evidence regarding the association between trust and objective or observer‐rated outcomes remains limited and inconsistent [23, 24], trust has been associated with patient‐reported outcomes, including subjective experiences such as patient satisfaction [19, 23, 25, 26, 27] and health‐related quality of life [19, 28, 29], as well as self‐reported health‐promoting behaviours [23]. Therefore, understanding the factors that influence the development of patient trust remains an important area of research.

Although trust in the physician–patient relationship has received increasing attention over the last two decades, most studies have focused on trust within established, long‐term relationships [30]. These studies suggest that patients tend to report higher levels of trust in physicians with whom they have an ongoing relationship [31]. However, patients must also place trust in clinicians they know relatively little about, and this trust may be shaped by first impressions and the initial interaction [32].

### 1.1 Specifics of a Newly Established Physician–Patient Relationship

The first encounter between a physician and a patient holds many specific characteristics. A mutual relationship has not yet been established, and the physician often lacks previous anamnestic data [33]. These are settings where patients typically seek care for a new or worsened symptom [34]. Newly arising health problems are often accompanied by patient concerns and expectations that may not be fully communicated or addressed during the clinical encounter [35]. Given the physician's usual effort to provide treatment or referral to another specialist as quickly as possible, patients may leave dissatisfied because of insufficient communication, a lack of personalised care [36], and insufficient time for additional patient questions [37]. All these circumstances place high demands on the level of interpersonal skills of physicians and on building mutual trust from the first moment of their encounter.

Only a very limited number of studies have examined trust in the context of the initial physician–patient encounter. Yet in many healthcare settings, patients are required to disclose sensitive information, accept recommendations, and engage in treatment before a long‐term relationship with a physician has been established. Understanding how trust develops under these circumstances is therefore important both for advancing theoretical understanding of patient trust and for informing communication practices during early clinical encounters. We know even less about the factors that shape patient trust before any contact has occurred. Therefore, the aim of this scoping review was to assess factors contributing to the development of patients' trust in newly established physician–patient relationships. Given the limited and heterogeneous nature of the available literature, a scoping review was considered the most appropriate approach to map the existing evidence and identify gaps for future research [38, 39].

## 2 Materials and Methods

This study is reported in accordance with the PRISMA Extension for Scoping Reviews (PRISMA‐ScR) guideline [40]. The full PRISMA‐ScR checklist is provided in Appendix A. A review protocol was not registered prior to conducting this scoping review, but it is available upon request from the first author.

A structured keyword search using the terms ‘trust AND (new OR first) AND (professional‐patient relationship OR physician–patient relationship OR therapeutic alliance)' was conducted in the online database PubMed, as this database provides the most comprehensive coverage of biomedical literature and health communication research relevant to our topic. The full search strategy is provided in Appendix B. The search yielded a total of 2825 studies published from January 1975 to 16 January 2026. After removing duplicates, 2818 unique studies remained for further review.

Studies were evaluated according to predetermined selection criteria. Studies were shortlisted if, upon reviewing the title and abstract, they met all the inclusion criteria: if they focused on the first encounter, examined physician–patient interaction, and included trust as either the primary variable or one of the variables assessed. Studies that concerned relationships that were already established (e.g., repeated patient visits to a general practitioner's clinic) or interactions with other healthcare professionals (e.g., nurse, physiotherapist, psychotherapist, etc.) were not considered eligible. In cases where doubts arose about whether the selection criteria were met (e.g., unclear from the abstract whether it was a first contact or not), the study was shortlisted and subjected to further evaluation. Title and abstract screening were conducted by the first author (BM) and two collaborating authors (Š.P., and J.Š.), who independently identified potentially eligible studies according to the inclusion and exclusion criteria. In cases of disagreement or uncertainty, the publication was retained for full‐text screening.

Based on the title and abstract assessment, 142 publications were identified for shortlisting. Subsequently, studies in languages other than English were excluded, reducing the selection to 137 publications. Publications that offered commentaries or reflections on the topic of trust in physician–patient interaction, as well as those not meeting the selection criteria, were excluded. Based on the final full‐text assessment conducted by the first author (BM), 10 studies were identified and included in the thematic analysis (Table 1).

**Table 1:** Studies included in the thematic analysis.

	Title	Authors	Year	Journal	Type of study	Methods	Sample characteristics	Location	Objective	Trust as primary outcome	Quality rating
1	Beliefs and distress about orofacial pain: patient journey through a specialist pain consultation [41].	C. Bonathan, et al.	2014	Journal of oral & facial pain and headache	Qualitative	Semi‐structured interviews before and after the first visit, written narratives after the visit.	12 patients with chronic orofacial pain; age range 26–73 years; 4 men, 8 women	Specialist orofacial pain clinic in London, United Kingdom	To explore patients’ understanding of their orofacial pain, as this is an under‐researched area despite emerging as a common aim of consultation.	No	Low risk
2	Building trust and rapport early in the new doctor‐patient relationship: a longitudinal qualitative study [42].	B. Dang et al.	2017	BMC Medical Education	Qualitative	Longitudinal, in‐person interviews across three time points (before the first visit, within two weeks after the first visit, and six to twelve months after the first visit).	21 patients newly entering care at an HIV clinic; age range 25–76 years; 19 men, 2 women; ethnically diverse sample	HIV clinic at the Michael E. DeBakey Veterans Affairs Medical Center in Houston, Texas, United States	To identify what patients see as the most critical elements for building trust and rapport from the outset	Yes	Low risk
3	Ask questions (ASQ): Implementation of a question prompt list communication intervention in a network of outpatient medical oncology clinics [43]	S. Eggly et al.	2023	Patient Education and Counselling	Quantitative	Descriptive single‐arm implementation design using the RE‐AIM framework, with patient‐reported surveys administered at three time points (baseline, pre‐visit, post‐visit)	81 patients with confirmed cancer diagnosis; 24 men, 57 women; participants varied in educational level and urban/rural background	Network of outpatient oncology clinics, Karmanos Cancer Institute, Michigan, United States	To assess the implementation and patient perceptions of the ASQ Question Prompt List across a network of oncology clinics using the RE‐AIM framework	No	High risk
4	‘I Missed My Other Oncologist’: Established Relationships as Barriers and Facilitators to Accessing CAR‐T and Autologous Transplantation[44]	Z. A. K. Frosch et al.	2025	Cancer Medicine	Qualitative	Semi‐structured interviews	40 patients with non‐Hodgkin lymphoma, Hodgkin lymphoma, or multiple myeloma eligible for or treated with ASCT/CAR‐T; age groups ranged from 18 to > 65 years; 20 men, 20 women; 45% identified as Black or African American	Multi‐site academic health system in Philadelphia, Pennsylvania, United States (including a safety net hospital)	To explore mechanisms by which oncologist continuity and the need to establish trust with a new provider influence access to ASCT/CAR‐T therapy, particularly in the context of health disparities	No	Low risk
5	What factors shape doctors trustworthiness? Patients' perspectives in the context of hypertension care in rural Tanzania [45].	K. Isangula et al.	2020	Rural and remote health	Qualitative	Interviews	34 patients receiving hypertension care in rural, low‐income Tanzania; age mostly 41–60 years; participants had varied educational attainment, most commonly primary or secondary education	12 health facilities in two predominantly rural districts of Shinyanga region, Tanzania	To examine factors that shape doctors’ trustworthiness in the context of hypertension care in rural low‐income Africa.	Yes	Moderate risk
6	Patient Characteristics and Experiences Associated With Trust in Specialist Physicians [46].	N. Keating et al.	2004	Archives of Internal Medicine	Quantitative	Picker‐Commonwealth Survey of Patient‐Centred Ambulatory Care with additional question to measure trust.	417 patients attending specialist physicians; 101 men, 316 women; age groups ranged from < 35 to > 65 years; predominantly White sample	Hospital‐based practices affiliated with a major academic medical centre in Boston, Massachusetts, United States	To examine how patients’ characteristics and experiences and related to trust in specialist physicians.	Yes	Low risk
7	The Role of Patient Research in Patient Trust in Their Physician [47].	L. Lu et al.	2019	Journal of Hand Surgery	Quantitative	General Self‐Efficacy short form (GSE‐6), Maximisation Short Form, Trust in Physician Form.	134 patients attending a hand surgery clinic; mean age 49.5 years; 69 men, 65 women; predominantly White sample with generally high educational attainment	Hand surgery clinic in a suburban academic medical centre in Redwood, California, United States	To investigate whether patient researching behaviour, maximising personality and general self‐efficacy were correlated with patient trust in their hand surgeon.	Yes	Moderate risk
8	Does being informed and feeling informed affect patients’ trust in their radiation oncologist? [48]	E. Smets et al.	2013	Patient Education and Counselling	Quantitative	Checklist after first consultation, follow‐up questionnaire one week prior to follow‐up consultation using 5‐item Trust in Physician Scale, 20‐item Trait‐subscale of State‐Trait Anxiety Inventory (STAI).	111 patients attending an initial consultation with a radiation oncologist; age range 28–86 years; 68 men, 42 women; predominantly Dutch sample	Radiotherapy department of the Academic Medical Center in Amsterdam, Netherlands	To investigate whether the content of information provided during initial consultation and radiation oncologists’ information giving behaviour predict patients’ trust in them.	Yes	Moderate risk
9	Chronic pain patients’ evaluations of consultations: A matter of high expectations or expectations unmet? [49]	C. M. Thompson et al.	2024	Patient Education and Counselling	Quantitative	Prospective study using pre‐ and post‐consultation surveys; development of self‐report Likert‐type measures assessing expectations, trust, satisfaction, and agreement with the physician.	200 patients presenting for an initial consultation for chronic musculoskeletal pain; mean age 52.0 years; 76 men, 119 women; predominantly White sample with varied educational attainment	A neuroscience institute in the Midwest United States, Illinois	To investigate which patient expectations are most often met, unmet, or exceeded	No	Moderate
10	Older Adults’ Experiences and Expectations of Doctor–Patient Interactions During Early Hospital Care [50]	G. Wells et al.	2025	Health Expectations	Qualitative	Semi‐structured, in‐depth interviews	20 hospitalised older adults aged 75–95 years with multimorbidity, polypharmacy, and/or frailty; 9 men, 11 women	Concord Repatriation General Hospital, Sydney, Australia	To investigate the perspectives of older patients, exploring their experiences and expectations during early hospital encounters	No	Moderate risk

Relevant data were extracted manually by the first author (BM) using a structured template that included study design, setting, sample characteristics, measures of trust, and main findings. Extracted data were cross‐checked with co‐authors (Š.P., and J.Š.) to ensure accuracy and resolve uncertainties in interpretation. Quantitative findings were first transformed into descriptive statements to enable their integration with qualitative evidence [51]. Thematic analysis was led by the first author (BM) and involved familiarisation with the data, generating initial codes, searching for potential themes, reviewing and refining these themes, defining and naming them, and finally producing the analytical narrative. The emerging themes and interpretation of findings were discussed with the co‐authors (Š.P., J.Š., and M.L.). The fourth author (M.L.) contributed to conceptual development, methodological supervision, and refinement of the analytical interpretation. The second and third authors (Š.P., and J.Š.) contributed to the screening and data collection process, particularly the parallel identification and assessment of potentially eligible studies according to the inclusion and exclusion criteria.

As the thematic analysis was conducted by a single author (BM) with a professional background in psychiatry, psychotherapy, and medical communication, the interpretation of the findings inevitably reflects this perspective. This expertise likely enhanced sensitivity to relational and communicative nuances in the physician–patient encounter, while potentially underemphasising other contextual or systemic factors. To enhance reflexivity and reduce the influence of a single interpretive perspective, the coding structure, emerging themes, and analytical narrative were discussed with the co‐authors (Š.P., J.Š., and M.L.) and refined through their feedback.

**Figure 1:**
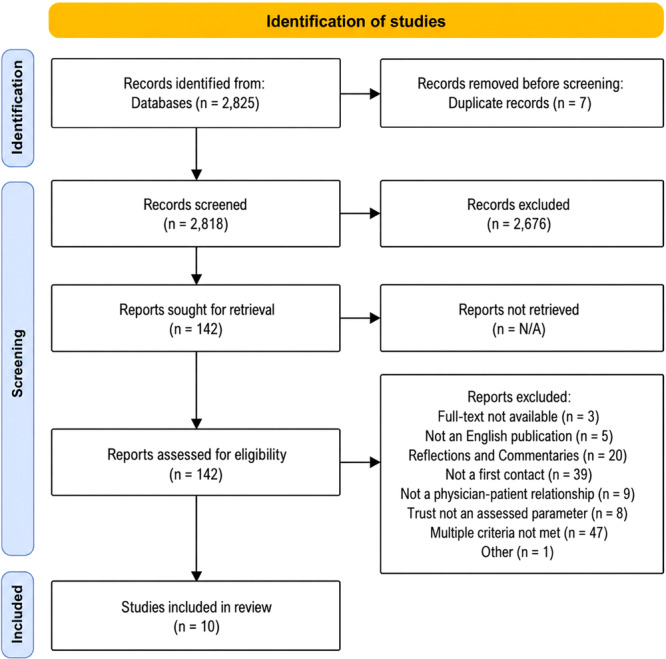
PRISMA flow diagram of study selection process.

Although formal risk of bias assessment is not a mandatory component of scoping reviews, we conducted an appraisal of study quality to enhance the transparency and robustness of our findings. A risk of bias assessment was conducted by the first author (BM) using the CASP (Critical Appraisal Skills Programme) Qualitative Research Checklist [52] for qualitative studies and the ROBINS‐I (Risk Of Bias In Non‐randomised Studies of Interventions) [53] tool for quantitative observational studies. The results of the appraisal were not used to exclude studies but were considered when interpreting the strength and limitations of the evidence. The study selection process is presented in Figure 1. The diagram was created using the PRISMA2020 R package and Shiny app [54].

## 3 Results

Ten studies were included in the review, comprising five qualitative and five quantitative designs. The studies were conducted across diverse countries (United States, United Kingdom, Australia, Tanzania and Netherlands) and predominantly focused on outpatient or ambulatory care settings, including specialty clinics and a radiotherapy department. In half of the studies, trust in the physician was a primary outcome, although the conceptualisation and measurement of trust varied considerably across designs. The studies also differed in terms of patient populations, ranging from specific ethnic groups to broader community samples. Across the included studies, several factors were suggested as associated with trust building during the initial physician–patient encounter, either positively or negatively.

### 3.1 Factors Positively Associated With Trust Building

Factors positively associated with trust building in the initial physician–patient encounter appear to cluster into three groups based on the phase in which they may influence trust: pre‐consultation, during consultation, and post‐consultation. Our analysis of the included studies indicated ten themes that may play a role in fostering trust within these three groups (Table 2). Within each category, themes are presented descriptively and do not reflect their relative importance, frequency, or strength of association across studies.

**Table 2:** Factors associated with trust building**.**

	Factors associated with trust building
Categories	Themes
Pre‐consultation phase	Physician reputation Recommendations and care coordination Previous positive experiences
During‐consultation phase	Physician's warm approach Perceived technical skills Information sharing and education Engaging patient participation Active listening and empathy (−) Limited opportunities to ask questions (−) Time constraints (−) Failure to meet patient expectations (−) Stigmatisation and inappropriate language
Post‐consultation phase	Sense of relief and recovery Apology for medical malpractice

*Note:* Themes identified in the included studies are presented according to the phase of consultation in which they appeared to influence trust (categories). Within each category, themes are presented descriptively and do not reflect their relative importance, frequency, or strength of association across studies. Factors negatively associated with trust are marked with (−).

#### 3.1.1 Pre‐Consultation Phase

Before the patient's initial visit to the physician, several factors may influence trust building:

- **Physician reputation:** Patient's trust may be shaped by the physician's standing within their community or social network. The perception of a positive reputation appears to facilitate trust [44], especially when the physician is known for possessing professional expertise and exceptional interpersonal skills [45]. Similarly, being examined by one of the leading physicians within a specific healthcare facility has been described as contributing to greater patient confidence in the care received [41].
- **Recommendations and care coordination:** Patients described that explicit recommendations from trusted healthcare professionals and clear communication between providers facilitated more rapid trust in new physicians [44]. Similarly, patients who received sufficient information from their referring physician about what to expect during a specialist consultation reported higher levels of trust in the specialist physician [46].
- **Previous positive experiences:** Patients who had prior positive experiences with healthcare appeared to report higher levels of trust in subsequent encounters [49, 50]. This effect was not limited to their own direct experiences. Trust could also be shaped indirectly, for example by witnessing satisfactory care provided to a close family member [50], or through positive experiences reported by others within the patient's social circle [44]. Reassuring personal research about a provider was described as another factor potentially supporting trust before the initial consultation [44].

#### 3.1.2 During‐Consultation Phase

The patient's initial impression of the attending physician may shape their experience of the consultation and influence the development of trust [42]. The included studies identified several factors that appeared to influence trust during the initial consultation:

- **Physician's warm approach:** Patients seemed to value a physician's warm and welcoming approach as an important factor in trust building [45]. They attributed meaning to initial greetings, non‐verbal communication such as positive facial expressions and respectful posture [45], as well as to the physician's displayed confidence [44]. Patients appeared to pay attention not only to the beginning of the consultation but also to its conclusion, where expressions of concern, wishes for a swift recovery, and an explicit invitation to return if problems persisted were described as potentially strengthening trust [45].
- **Perceived technical skills:** Patients' belief that their new physician was prepared for the initial meeting appeared to support trust [44]. Perceived technical competence, such as taking a detailed medical history, asking relevant questions, conducting a thorough physical examination, ordering appropriate diagnostic tests, and proposing suitable treatment, was described as an important marker of professional expertise [45]. Demonstrating a high level of knowledge further reinforced this perception [44], and patients' belief that their physician was skilled and perceptive seemed to be associated with greater trust [50]. Addressing the presenting complaint was described as a highly valued aspect of the first consultation [50].
- **Information sharing and education:** Patients expressed a wish to understand the reasoning behind recommended examinations and treatment decisions. They emphasised the importance of reviewing results together with the physician and receiving clear guidance in interpreting them, rather than being reassured by a brief statement that ‘laboratory results were fine.’ [42] A clear and understandable explanation of the diagnosis [41], information about potential side effects of prescribed medications [45], and feeling adequately informed overall appeared to be associated with higher levels of trust [46]. At the end of the visit, patients seemed to value guidance on when to seek further care or what steps to take if symptoms persisted or worsened [45, 46]. However, being overwhelmed by excessive information at the outset could negatively influence trust [44].
- **Engaging patient participation:** Greater trust was described when physicians actively encouraged patient participation in the treatment process [42, 45]. Patients wished to be asked about their needs and involved in discussions and shared decision‐making [42, 45]. Importantly, patients appeared to be comfortable with physicians making the final medical decisions, as long as those decisions were clearly explained to them [50]. In contrast, perceiving a lack of involvement seemed to be associated with dissatisfaction and reduced trust [50]. Encouraging patients to ask questions, for example through the use of Question Prompt List (QPL) brochures, may be associated with greater engagement and could potentially relate to higher perceived trust, although this relationship should be interpreted with caution [43].
- **Active listening and empathy:** Patients consistently highlighted the importance of feeling heard, understood, and treated as individuals [41, 50]. Strong, individualised communication accompanied by empathy appeared to be associated with higher levels of trust [44], and survey data similarly linked enhanced trust with physicians listening to everything ‘that was on the patient's mind.’ [46] In contrast, trust could be diminished when physicians appeared distracted, for example by focusing on the computer while patients were describing their concerns [45]. Expressions of gentleness, care, compassion [45], reassurance, and hope [42, 50] were described as potentially strengthening trust, whereas a lack of sympathy or ignoring signs of emotional distress might have had the opposite effect [45, 48].

#### 3.1.3 Post‐Consultation Phase

The patient's trust in the physician may continue to evolve even after the initial consultation. This trust might be reinforced when the procedures recommended by the physician are perceived as leading to:

- **Sense of relief and recovery:** Trust was described as strengthened when patients experienced relief, alleviation of symptoms, or recovery following the physician's recommendations [45]. These positive outcomes seemed to further support the patient's confidence in their physician.
- **Apology for medical malpractice:** If medical malpractice occurred, an apology from the physician appeared to be an important factor in maintaining or restoring trust [45].

### 3.2 Factors Negatively Associated With Trust Building

To gain insights into how trust building during the initial patient visit is influenced, it is also important to consider factors that may negatively impact this process. Drawing from the included studies, four themes were identified that appeared to adversely affect trust in physicians (Table 2). All negatively associated factors were reported in relation to the consultation itself, while no negative influences were identified in the pre‐ or post‐consultation phases.

#### 3.2.1 During‐Consultation Phase

- **Limited opportunities to ask questions:** Trust was described as diminished when physicians did not actively ask whether patients had questions [45], discouraged inquiries, or left questions unanswered [48].
- **Time constraints:** The perception of limited time for physician preparation and mutual conversation was associated with reduced trust [45]. Patients reported diminished confidence when specialists appeared unprepared or frequently searched for information on the computer during the visit [48]. Feelings of time pressure during the consultation negatively influenced trust, whereas having sufficient time to explain the reasons for the visit helped maintain confidence [46]. Similarly, rapid prescription of medication without a comprehensive assessment of the patient's condition was described as undermining trust [45].
- **Failure to meet patient expectations:** Trust was described as decreasing when physicians failed to meet patients' expectations, particularly when patients came to the consultation with specific hopes that were not fulfilled, such as expectations for specific investigations, treatments, or explanations that fell outside the physician's role or the facility's capabilities [41]. However, another study did not find a negative association between unmet expectations and interactional outcomes, including patient satisfaction, trust in the physician, or agreement with the physician [49]. Thus, the included studies reported mixed findings regarding the association between unmet expectations and trust‐related outcomes.
- **Stigmatisation and inappropriate language:** Stigmatising attitudes or assumptions held by physicians, whether conscious or unconscious, were described as undermining trust [42]. Such stigma could be reflected in physicians' behaviours, including allocating less time to patients, lowering expectations of adherence, or limiting referrals to preventive or specialty care [42]. Patients who perceived stigmatising attitudes or inappropriate language from their physicians reported reduced trust [45], and experiences of verbal harassment were likewise described as detrimental to the physician–patient relationship [45].

### 3.3 Contextual Patterns and Potential Boundary Conditions

The included studies did not allow for a formal assessment of moderators, as they differed substantially in design, clinical setting, patient population, and measurement of trust. Nevertheless, several contextual patterns emerged that may help explain when and how the identified factors shape trust in newly established physician–patient relationships.

First, patients' previous experiences with healthcare appeared to influence their readiness to trust a new physician. Positive prior experiences, recommendations from trusted professionals, and reassuring information from others were described as facilitating trust [44, 45, 49, 50], whereas previous experiences of dismissal, stigma, insufficient explanation, or low satisfaction with prior care may increase patients' caution in subsequent encounters [41, 42, 45, 49]. Second, uncertainty, vulnerability, anxiety, or the need for validation appeared to shape which physician behaviours were especially important for trust formation. In studies of chronic orofacial pain and new HIV care, patients entered the consultation with worry, vulnerability, or a strong need to feel understood, suggesting that clear explanations, validation, and empathic communication may be particularly important in these contexts [41, 42]. This pattern was partly supported by quantitative evidence showing a negative bivariate association between trait anxiety and trust [48]. Finally, the relative importance of trust‐building factors appeared to vary across clinical contexts: validation and explanation were prominent in chronic pain settings [41, 49], whereas continuity of care during referral to a new specialist, professional recommendations, and coordination between care teams were especially salient in specialised oncology care [44]. Although several studies reported demographic characteristics such as age, gender, education, race, ethnicity, or socioeconomic status, these variables were not examined consistently enough to support conclusions about their moderating role.

## 4 Discussion

This scoping review suggests that trust in newly established physician–patient relationships is shaped throughout the initial care trajectory, across the pre‐consultation context, the quality of the consultation itself, and post‐consultation outcomes such as symptom relief, recovery, or responses to adverse events. In the absence of an established relationship, patients may rely on early cues of competence, benevolence, professionalism, and care coordination to decide whether a new physician is trustworthy.

Our findings are broadly consistent with existing conceptualisations of physician trust, particularly the domains of technical competence, interpersonal competence, and agency described by Thom et al. [4] Factors related to physicians' perceived clinical expertise, communication style, empathy, and commitment to patients' welfare were all represented in the included studies, suggesting that the theoretical dimensions of trust identified in established physician–patient relationships may also be relevant during first encounters. At the same time, our findings indicate that newly established relationships may place particular emphasis on indirect and rapidly available indicators of trustworthiness, such as reputation, professional recommendations, and care coordination. The contextual patterns identified in this review further suggest that trust formation during first encounters may be shaped by potential confounders, moderators, or boundary conditions, including patients' prior healthcare experiences, current uncertainty or vulnerability, and the clinical context of care. These factors may influence how patients interpret physicians' behaviours during the consultation and may help explain why the same trust‐building behaviour may carry different weight for different patients. Given the limited and heterogeneous evidence base, these patterns should be interpreted as tentative rather than as established explanatory mechanisms.

The results of our scoping review are consistent with findings from studies focused on trust building in general or in long‐term physician–patient relationships [55, 56, 57, 58]. Greene et al. [58] further highlight the importance of an individual approach to patients and of valuing their experiences. Beyond the factors we have identified, patients' trust is strengthened when they feel that their concerns are taken seriously [59]. Hogikyan et al. [60] also emphasise the role of patients' previous experiences with physicians' approach and the quality of healthcare services received in the past [60]. Langerman et al. [61] further suggest that previous negative experiences likely not only influence trust in a new physician but also contribute to overall distrust in the healthcare system. In addition to the reputation, trust is also influenced by interactions between the physician and other patients or healthcare team members that the patient witnesses [60]. Taken together, these studies point to similarities with our findings, particularly the role of communication, prior positive experiences and the physician's reputation. At the same time, there are differences: while long‐term trust is shaped by accumulated patient experiences, our review suggests that initial trust relies more heavily on immediate cues such as communication style or the physician's management of time during the first encounter.

It is noteworthy that the reviewed studies did not emphasise the absolute length of the consultation as a determinant of trust. Instead, patients described that their trust was more strongly influenced by a subjective sense of time pressure or insufficient opportunity for conversation with the physician. These findings are supported by other studies [59, 62]. Braddock and Snyder [63] in their review recommend spending ‘adequate time’ with the patient based on their needs. They suggest that rather than the length of consultation alone (quantity of time), the crucial factor is how the time is utilised (quality of time).

One of the factors that seemed to be positively associated with patients' trust was physicians' willingness to apologise for medical errors. However, existing evidence suggests that medical errors are not always disclosed in practice [64]. This is due to physicians' fear of losing patient trust, the patient's emotional reaction, or lawsuits for medical malpractice [64]. Nonetheless, Mazor et al. [65] argue that disclosure of medical errors does not necessarily carry negative consequences for physicians.

The limited number of studies available for inclusion in the thematic analysis is both a constraint on the synthesis and an indication of the current state of the literature. It suggests that trust formation during the first physician–patient encounter has so far received limited research attention and that empirical data in this area remain scarce. Consequently, the findings should be interpreted as preliminary and indicative rather than definitive. The search strategy may also have excluded relevant studies. The use of specific keywords, such as ‘new’ and ‘first,’ might have excluded studies examining initial physician–patient interactions that did not explicitly use these terms. For example, it is possible that studies conducted in settings such as emergency or ambulatory care, where a first encounter may be implicit, were not retrieved. Similarly, the term ‘doctor–patient relationship’ was not included in the search strategy; however, as ‘physician–patient relationship’ is the predominant indexing term in the databases searched, we believe this omission had minimal impact on the comprehensiveness of our search. The literature search was also restricted to PubMed, which may have resulted in the omission of studies indexed in other databases. Only one eligible study examined first physician–patient encounters in the hospital setting. Further research is therefore needed to explore trust‐building dynamics in hospital settings and to provide a more comprehensive understanding of trust formation across different healthcare contexts. Another limitation is the substantial heterogeneity across the included studies. The strength of the conclusions is constrained by the study designs, and the findings should therefore be interpreted as indicative associations rather than causal relationships. This heterogeneity arose not only from methodological differences, including qualitative versus quantitative approaches and the use of diverse instruments to measure trust, but also from varying conceptualisations of the construct itself and from differences in demographic characteristics and contextual conditions across study populations and healthcare settings. Such variability may have contributed to potential bias through overgeneralisation and the possible omission of context‐specific factors influencing the establishment of trust. Several key review steps, including full‐text screening, data extraction, risk‐of‐bias assessment, and the thematic analysis, were primarily led by the first author (B.M.); therefore, the possibility of reviewer or interpretive bias cannot be fully excluded. However, extracted data, emerging themes, and the analytical interpretation were discussed with co‐authors (Š.P., J.Š., and M.L.) and refined through their feedback. The risk of bias assessment revealed varying levels of bias across the included studies. Importantly, only four of the ten included studies were rated as having low risk of bias, whereas five were rated as moderate risk and one as high risk. This uneven evidentiary base limits the certainty of the synthesis, particularly for themes supported by single studies or by studies with moderate or high risk of bias. Qualitative studies generally demonstrated low to moderate risk of bias, primarily due to the comprehensive exploration of participant perspectives and robust data collection methods, although some studies lacked explicit discussion of potential biases. Quantitative studies showed low to high risk of bias, particularly in areas concerning confounding factors and measurement of outcomes. Several studies also relied on self‐reported measures, which may introduce subjective bias. The findings should therefore be interpreted as preliminary and hypothesis‐generating rather than as definitive conclusions about trust formation. As in all reviews, the possibility of publication bias must be considered as well, since studies with non‐significant or null findings are less likely to be published.

## 5 Conclusion

This scoping review examined the dynamics of trust building between physicians and patients, exploring factors that may positively or negatively impact this crucial point in physician–patient relationship. The results of this scoping review appear to align with those of previously published studies examining trust in the context of a long‐term physician–patient relationship, with some additional factors potentially specific to initial encounters identified. Key elements identified in our review include the physician's reputation, communication skills, a compassionate approach, technical proficiency, information sharing, and post‐consultation outcomes such as symptom relief.

Trust plays a significant role in patients' active participation and adherence, and it therefore seems important to seek ways to strengthen it. To support patient trust, physicians may benefit from building a positive professional reputation not only by their interactions with patients but also by the ways in which they interact with colleagues. During the encounter, physicians could enhance their trustworthiness by actively addressing patients' questions and uncertainties. It may also be useful to actively enquire about patients' expectations and clearly communicate healthcare limitations to avoid the risk of patient disappointment from unmet expectations. In the context of time constraints commonly experienced by physicians, it could be appropriate to communicate the allocated time for mutual engagement and collaboratively schedule examinations with the patient based on the available time. During subsequent follow‐up, helping patients recognise the effects of therapy may further contribute to strengthening their trust.

Factors identified in this study (communication skills, active listening, expressing empathy, support for patient participation, etc.) represent areas that could be developed through appropriate educational approaches. Incorporating educational courses aimed at developing these interpersonal skills into the curricula of future and current physicians has the potential to indirectly increase patient trust and thus support their healing process.

## Patient or Public Contribution

This review synthesises findings from studies that explicitly report patients' perspectives on trust in newly established physician–patient relationships. While patients were not directly involved in the design, conduct, or interpretation of this review, patient‐reported experiences formed the primary source of evidence and informed the understanding of trust development in clinical practice.

## Author Contributions

**Barbora Mechúrová:** conceptualisation, methodology, formal analysis, investigation, writing – original draft. **Šárka Pomichálková:** investigation. **Janka Šefranková:** investigation. **Martin Loučka:** conceptualisation, methodology, supervision, writing – review and editing. This manuscript has not been published or submitted elsewhere. All authors have contributed significantly, and all authors agree with the content of the manuscript.

## Funding

The authors have nothing to report.

## Ethics Statement

The authors have nothing to report.

## Consent

The authors have nothing to report.

## Conflicts of Interest

The authors declare no conflicts of interest.

## Supporting information


Supporting File 1

Supporting File 2

## Data Availability

The data that support the findings of this study are available from the corresponding author upon reasonable request.
