# Supplementary material for: Building Trust in a Newly Established Physician–Patient Relationship: A Scoping Review
Source: Health Expect. 2026 Jul 27;29(4):e70788. doi: 10.1111/hex.70788 (PMC13407324; doi:10.1111/hex.70788)
Supplement: Supplementary file 2 — Supporting File 2 [file HEX-29-e70788-s001.docx]

**Search strategy:**

("trust"[MeSH Terms] OR "trust"[All Fields] OR "trusted"[All Fields] OR "trusting"[All Fields] OR "trusts"[All Fields] OR "trust s"[All Fields] OR "trustful"[All Fields]) AND ("new"[All Fields] OR ("first"[All Fields] OR "firsts"[All Fields])) AND ("professional patient relations"[MeSH Terms] OR ("professional patient"[All Fields] AND "relations"[All Fields]) OR "professional patient relations"[All Fields] OR ("professional"[All Fields] AND "patient"[All Fields] AND "relationship"[All Fields]) OR "professional patient relationship"[All Fields] OR ("physician patient relations"[MeSH Terms] OR ("physician patient"[All Fields] AND "relations"[All Fields]) OR "physician patient relations"[All Fields] OR ("physician"[All Fields] AND "patient"[All Fields] AND "relationship"[All Fields]) OR "physician patient relationship"[All Fields]) OR ("therapeutic alliance"[MeSH Terms] OR ("therapeutic"[All Fields] AND "alliance"[All Fields]) OR "therapeutic alliance"[All Fields]))
